# Comparison of Multipulse Laser Vaporesection versus Plasmakinetic Resection for Treatment of Benign Prostate Obstruction

**DOI:** 10.1038/s41598-019-42903-6

**Published:** 2019-04-23

**Authors:** Fu-Shun Hsu, Chen-Wei Chou, Hong-Chiang Chang, Yuan-Po Tu, Shing-Jia Sha, Huang-Hsin Chung, Kuo-How Huang

**Affiliations:** 1 Department of Urology, New Taipei City Hospital, New Taipei City, Taiwan; 2 Graduate Institute of Clinical Medicine, National Taiwan University College of Medicine, Taipei, Taiwan; 3 Department of Urology, National Taiwan University Hospital, Taipei, Taiwan; 4 Shu-Tien Urology Ophthalmology Clinic, Taipei, Taiwan; 5 Department of Pathology, New Taipei City Hospital, New Taipei City, Taiwan

**Keywords:** Prostate, Thermoelectric devices and materials

## Abstract

We aimed to compare the efficacy and safety of Multipulse laser vaporesection of the prostate (MPVP) versus plasmakinetic resection of the prostate (PKRP) for treatment of patients with benign prostate obstruction (BPO) in a prospective trial. From January 2016 to April 2017, a total of 144 patients were included in the cohort study, of whom 73 patients underwent MPVP and 71 underwent PKRP. All patients received pre-operative evaluation and followed up at 1, 3, 6 and 12 months postoperatively. Baseline characteristics, perioperative data and postoperative outcomes were compared. Early (within 30 days postoperatively) and late complications were also recorded. Preoperative data, including age, prostate volume, international prostate symptom score (IPSS), International Index of Erectile Function Questionnaires (IIEF-5), the rate of anticoagulants use, Charlson comorbidity index were similar in two groups. Peri-operative parameters, including the rate of transfusion, and decrease in hemoglobin level were comparable. The operative time, the duration of catheterization and length of hospital stay were significantly shorter in the MPVP group. The voiding parameters and the quality-of-life scores (QoL) improved significantly in both groups postoperatively. There was a significantly difference in QoL at 1-year in the MPVP group (*p* < 0.001), under mixed model analysis with random effect and Bonferroni correction. There were no significant differences in improvement of IPSS, Qmax, IIEF-5, residual prostate volume ratio and PSA level reduction at the 1-year follow-up. MPVP was significantly superior to PKRP in terms of a reduction in overall complication rate (21.9% vs 45.0%, *p* = 0.004). Both treatments led to comparable symptomatic improvements. MPVP demonstrates satisfactory efficiency, shorter catheterization time and shorter hospital stay. Our data revealed that MPVP may be a promising technique which is safe and favorable alternative for patients with BPO.

## Introduction

Benign prostate obstruction can be treated with a range of laser treatments using different laser systems and applications. Transurethral laser treatment is considered to be an alternative treatment to transurethral resection of the prostate (TURP)^1^. In the past decade, different kinds of laser systems are used to produce various tissue ablations, such as coagulation, vaporization or resection and/or enucleation, in the treatment of BPO^2–4^.

Previous studies have compared the outcomes between TURP and transurethral laser treatment. Compared to TURP, thulium laser prostatectomy reduced blood loss, shorten catheterization time and hospital stay, and yielded better efficacy^3^. The diode laser was reported to combine high tissue ablation capacity and good hemostatic properties^5,6^. Tan *et al*. indicated that diode laser was better than thulium laser for prostate vaporesection because of its shorter catheterization time^7^. The re-operation rate for diode laser treatment was higher compared to that for 532 nm greenlight laser, photoselective vaporization of the prostate^8^. Growing evidence on safety and efficacy regarding these laser treatments have been revealed, all these available laser treatments have individual advantages and disadvantages.

A recently introduced double laser combination system, the Multipulse Tm + 1470 laser system (Asclepion, Jena, Germany), has been applied for BPO treatment. The device is a combination of a Thulium:YAG laser that emits a wavelength of 1940 nm and a near infrared diode laser module that emits a wavelength of 1470 nm. The total laser power is up to 150 Watts and transmitted through end-firing optical fibers. The Multipulse laser might represent a similar resection efficacy as thulium and optimization of hemostatic efficacy deriving from the combination with the second wavelength. The data of safety and efficacy comparing Multipulse laser vaporesection of prostate (MPVP) and plasmakinetic resection of the prostate (PKRP) are still lacking.

In this study, we prospectively assessed the clinical outcome of MPVP versus PKRP with a 1-year follow-up. To our knowledge, this study is the first report on the feasibility and efficacy of Multipulse Tm + 1470 laser system for treatment of BPO.

## Methods and Materials

### Study design and patients enrollment

This study is a prospective, single-center, clinical observation to compare outcomes of MPVP versus PKRP. Between January 2016 and April 2017, a total of 160 patients with symptomatic BPO underwent treatment with the Multipulse Tm + 1470 laser system (80 cases) or the PKRP (80 cases). Inclusion criteria were patient age older than 50 years, maximum flow rate (Qmax) < 15 mL/second and the International Prostate Symptom Score (IPSS) ≥ 10. All subjects received quality-of-life questionnaire (QoL) and transrectal ultrasound of the prostate (TRUS) preoperatively. TRUS biopsies were performed before surgery in cases of suspicious malignancy. Patients with prostate cancer, bladder cancer, previous transurethral surgery, interstitial cystitis and neurogenic bladder were excluded. The use of anticoagulants or platelet aggregation inhibitors and urinary retention under catheterization was not a criterion for study exclusion. Patients taking anticoagulants or platelet aggregation inhibitors were requested to discontinue the use of these drugs three days before operation. The Charlson comorbidity index (CCI) was used to measure underlying comorbid disease status^9^. This study was approved by the institutional review board at New Taipei City Hospital (No. 105005-E). We have registered our study in WHO primary clinical trial registry on February, 21, 2019 and obtained the registration number (ChiCTR1900021449). All methods were performed in accordance with the relevant guidelines and regulations of the institution. The written informed consent forms from all subjects who met the inclusion criteria were obtained. A total of 144 patients were included in the study (73 MPVPs and 71 PKRPs), all signed an informed consent. Eight patients lost follow-up (3 MPVPs and 5 PKRPs).

### Surgical technique

The MultiPulse Tm + 1470 laser system combines a Thulium:YAG laser emitting at a wavelength of 1940 nm and a near infrared diode laser module emitting at a wavelength of 1470 nm. The laser power can reach 150 Watts (120 Watts Thulium:YAG laser + 30 Watts diode laser) and is then transmitted through end-firing optical fibers.

All patients were placed in the lithotomy position under spinal or general mask anesthesia. Two experienced urologists (F.S.H. and C.W.C.) carried out all procedures in this study. Each had performed over 800 cases with transurethral resection of prostate with either laser or electrocautery. We used a 26-Fr resectoscope sheath (Olympus, Tokyo, Japan) coupled with either laserscope or resectoscope for both groups. Continuous normal saline irrigation of the operative field was brought throughout the operation. The energy setting of MPVP during surgery was within the range: Thulium:YAG laser power 80–120 Watts and diode laser power 20–30 Watts. The vaporesection procedure began with incision manner at 1 and 11 o’clock of the bladder neck, to the level of the verumontanum. The incision depth was continued to the prostate capsule. The vapoincision lines were then created at the 5 and 7 o’clock position to separate the right, median and left lobes. The three lobes were enucleated in sequence, and pushed into the bladder. Tissue was vaporized or vapoincised sufficiently so that small pieces could be removed through the laserscope by a balloon evacuator. Otherwise, intravesical morcellation by tissue morcellator was performed to remove the resected tissue.

In the PKRP group, the resection power was 160 Watts and the electric coagulation was set at 80 Watts. A reflux plasma prostate resectoscope (Olympus, Tokyo, Japan) was used. A longitudinal groove was first made at 5 and 7 o’clock, deep into the surgical capsule, from the bladder neck toward the proximal of the verumontanum. The bilateral lobes and median lobe were resected in sequence, and the procedure was finished by trimming the apex. The resected chips were washed out by an Ellick balloon evacuator. At the end of both procedures, a 22-Fr triple-lumen catheter was placed into the bladder, and continuous irrigation of the bladder with saline was set up in all patients.

### Outcome measurement

The primary outcome measurement was IPSS at 12 months. The secondary outcome measurements were Qmax and QoL. The following parameters were assessed preoperatively and at 1-, 3-, 6- and 12-months intervals after operation: IPSS, International Index of Erectile Function Questionnaire (IIEF-5), Qmax, postvoid residual (PVR), QoL, PSA and prostate volume. The hemoglobin difference before surgery and the day after the surgery was documented. The operative time, catheterization time and hospital stay between two groups were compared.

The peri- and postoperative complications, classified using the Uro-Clavien-Dindo classification^9,10^, including blood transfusion, prolonged urinary infection, dysuria, prolonged hematuria, re-catheterization, re-operation, incontinence, urethra stricture and scrotal edema were recorded and compared. With consideration of safety issue, any adverse events (AEs) ≥ Grade III would be immediately reported and well inspected.

### Statistical analysis

All data in this study are presented as mean ± standard deviation. Stata Corp Stata 15 was used for statistical analysis. The Fisher’s exact test was applied to compare categorical variables; and the Student’s t-test was used to compare quantitative variables between the two treatment groups. A mixed model with random effect and Bonferroni correction was applied to compare IPSS, QoL, Qmax, and PVR between two groups. A two-sided *p*-value of <0.05 was considered statistically significant.

## Results

### Baseline and perioperative parameters

A total of 144 patients (MPVP: 73, PKRP: 71) were included in this study. The demographic data of the patients are shown in Table 1. The mean age of the patients in the MPVP and PKRP groups was 68.8 ± 8.3 and 69.9 ± 9.2 years, respectively (*p* = 0.438). The mean prostate volume (57.0 vs. 60.2 mL), PSA (3.8 vs. 4.2 ng/mL), IPSS (21.7 vs. 22.5), IIEF-5 (5.1 vs. 5.9), percent of indwelling catheter (13.7% vs. 16.9%), and the Charlson comorbidity index^9^ (CCI, 1.47 vs. 1.44) were similar in two groups, MPVP versus PKRP (all *p* > 0.05).

**Table 1:** Preoperative patients’ demographic characteristics.

	MPVP (*n* = 73)	PKRP (*n* = 71)	*p* value
Age	68.8 ± 8.3	69.9 ± 9.2	0.438
Prostate volume (mL)	57.0 ± 18.4	60.2 ± 20.0	0.318
IPSS	21.7 ± 6.4	22.5 ± 6.8	0.447
Qmax (mL/sec)	7.9 ± 4.1	7.1 ± 4.9	0.308
QoL	5.1 ± 0.7	5.1 ± 0.7	0.699
PVR volume (mL)	89.8 ± 10.8	94.3 ± 99.0	0.791
PSA (ng/mL)	3.8 ± 2.5	4.2 ± 2.8	0.321
IIEF-5	5.1 ± 7.6	5.9 ± 7.9	0.656
Anticoagulants use	21 (32.8%)	25 (35.2%)	0.476
Indwelling catheter	10 (13.7%)	12 (16.9%)	0.648
Prostate stones > 5 mm	20 (30.1%)	16 (22.5%)	0.566
Diabetes	27 (37.0%)	25 (35.2%)	0.863
Charlson comorbidity index	1.47 ± 1.92	1.44 ± 1.34	0.916

MPVP = Multipulse laser vaporesection of the prostate, PKRP = plasmakinetic resection of the prostate, IPSS = international prostate symptom score, Qmax = maximum flow rate, QoL = the quality-of-life scores, PVR = postvoid residual, IIEF-5 = International Index of Erectile Function Questionnaires.

Continuous variables are shown as the mean ± standard deviation.

Categorical variables are shown as the number (percent).

The perioperative parameters are listed in Table 2. There were no significant differences in the rate of transfusion, and decrease of hemoglobin level between two groups. However, compared with the PKRP group, patients in the MPVP group had shorter operative time (30.9 ± 10.3 vs. 36.4 ± 13.8 minutes, *p* = 0.008), shorter catheterization time (21.3 ± 4.6 vs. 37.4 ± 15.9 hours, *P* < 0.001), and shorter hospital stay (2.6 ± 1.0 vs. 3.6 ± 1.3 days, *p* < 0.001). The weight of resected adenoma was heavier in PKRP group compared to those in MPVP group (11.0 ± 8.0 vs. 20.6 ± 10.5 gm, *p* < 0.001).

**Table 2:** Perioperative parameters (MPVP vs. PKRP).

	MPVP (*n* = 73)	PKRP (*n* = 71)	*p* value
Operative time (min)*	30.9 ± 10.3	36.4 ± 13.8	0.008
Catheterization time (hr)*	21.3 ± 4.6	37.4 ± 15.9	< 0.001
Hospital stay (day)*	2.6 ± 1.0	3.6 ± 1.3	< 0.001
Resected adenoma weight*	11.0 ± 8.0	20.6 ± 10.5	< 0.001
Hemoglobin drop (gm/dl)	0.43 ± 0.66	0.53 ± 0.82	0.418
Transfusion	0	2 (2.8%)	0.246
Concomitant cystolithotripsy	4 (5.5%)	3 (4.2%)	1.000
Prostate cancer	0	0	N/A

*Statistically significant.

### Functional outcomes

**Figure 1:**
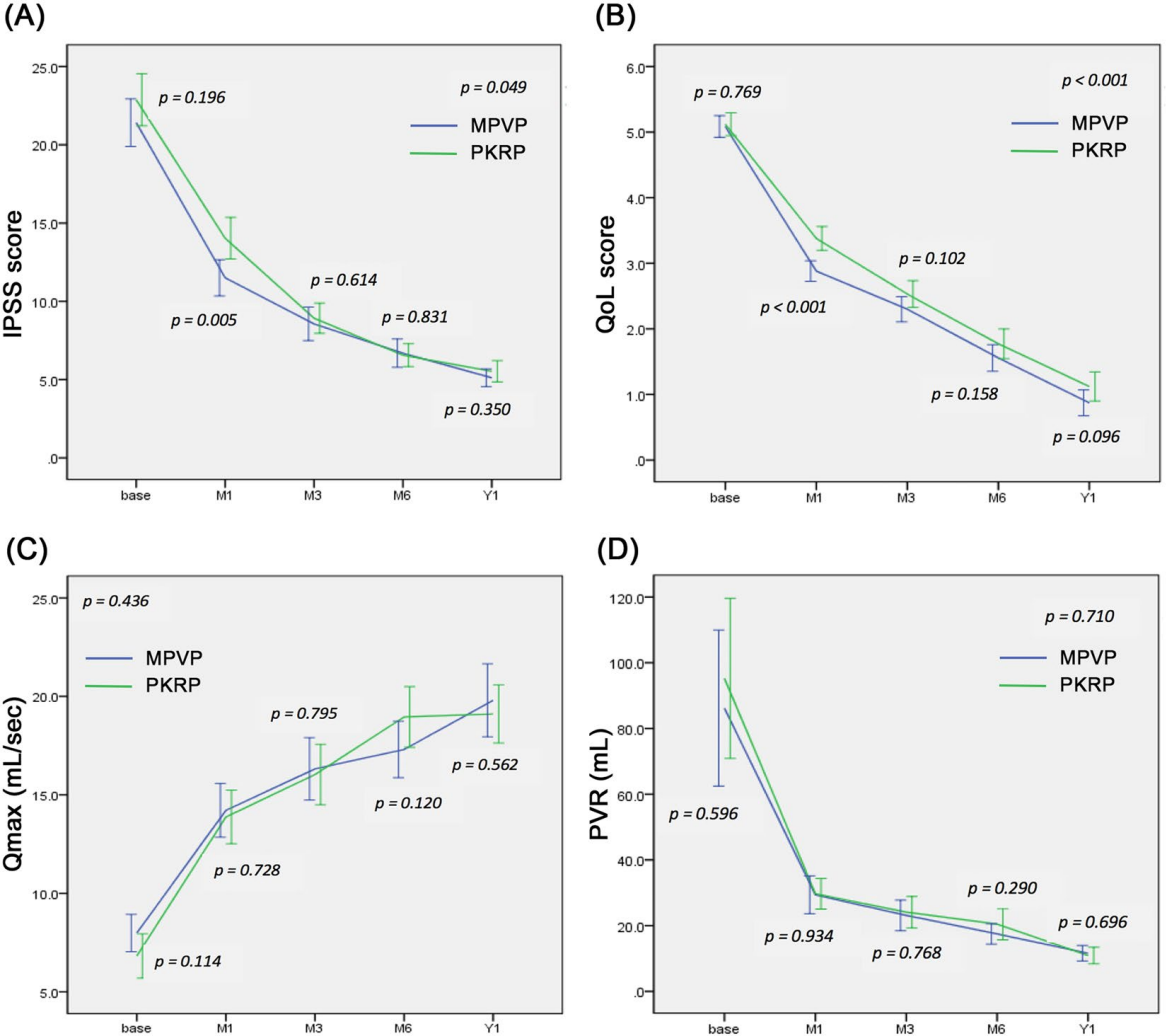
Outcomes following treatment with the MultiPulse laser (MPVP) or plasmakinetic resection of the prostate (PKRP). (**A**) International Prostate Symptom Score (IPSS), (**B**) quality-of-life index (QoL), (**C**) maximum flow rate (Qmax) and (**D**) postvoid residual urine (PVR). *P*-value of mixed model with random effect and Bonferroni correction was mentioned at the corner of each graph. Base: preoperative data, M1: 1-month, M3: 3-month, M6: 6-month, Y1: 1-year follow-up.

The improvement of functional outcomes is shown in Table 3 and Fig. 1. At the 12-month follow-up, the voiding parameters (IPSS, Qmax and PVR), IIEF-5 and QoL improved significantly compared with pre-operative data. Compared with PKRP group, the improvement of IPSS, QoL, Qmax and PVR in patients in MPVP group was not significantly different at 12 months post-operatively (*p* = 0.350, 0.096, 0.562 and 0.696, respectively). However, under mixed model analysis with random effect and Bonferroni correction, MPVP group was superior to PKRP group in IPSS and QoL (*p* = 0.049 and <0.001, respectively).

**Table 3:** Improvement of functional outcomes and PSA level at 1 year after surgery (MPVP vs. PKRP).

	MPVP preoperative	MPVP 1 year	PKRP preoperative	PKRP 1 year	*p* value MPVP vs PKRP (1 year)	*p* value Corrected (1 year)**
	(*n* = 70)		(*n* = 66)			
IPSS**	22.0 ± 6.3	5.7 ± 3.0*	21.9 ± 6.8	6.1 ± 3.1*	0.350	0.049
QoL**	5.1 ± 0.7	1.1 ± 0.9*	5.2 ± 0.7	1.8 ± 1.2*	0.096	< 0.001
Qmax (mL/sec)**	8.8 ± 5.7	18.7 ± 8.7*	7.7 ± 5.8	17.4 ± 6.5*	0.562	0.436
PVR (mL)**	89.8 ± 105.5	10.7 ± 10.8*	104.9 ± 113.5	15.1 ± 10.9*	0.696	0.710
IIEF-5	4.2 ± 7.1	11.5 ± 9.7*	4.6 ± 6.9	10.5 ± 9.0*	0.706	
PSA (ng/mL)	4.0 ± 2.8	2.3 ± 1.9*	4.3 ± 2.9	2.3 ± 1.8*	0.910	
Prostate volume (mL)	60.1 ± 22.3	23.6 ± 9.9	64.6 ± 26.5	28.0 ± 10.1	0.023	
Residual prostate volume ratio at 1 yr***		0.41 ± 0.15		0.47 ± 0.18	0.071	

*p < 0.001; *p*-value of *p*arameters at 1 year compared to preoperative data.

**Improvement of IPSS, QoL, Qmax, and PVR between two groups was analyzed by mixed model with random effect and Bonferroni correction, *P*-value of < 0.05 was considered statistically significant.

***Residual prostate volume ratio at 1 yr = residual prostate volume at 1-year/preoperative prostate volume.

### Adverse events

The postoperative complications were classified as early stage (within 30 days) and late stage (after one month) and summarized in Table 4. We used the Uro-Clavien-Dindo classification to evaluate treatment-related complications^10,11^. Overall, 78.1% of MPVP group and 55.0% of PKRP group were free of any treatment-related adverse event (AE, *p* = 0.004).

**Table 4:** Early (first 30 postoperative days) and late complications by the Uro-Clavien-Dindo classification system.

	MPVP (*n* = 73)	PKRP (*n* = 71)	*p* value
Overall complications*	16 (21.9%)	32 (45.0%)	0.004
Grade I			
UTI with bacteria cultured	5 (6.8%)	9 (12.6%)	0.272
Urinary incontinence	1 (1.4%)	4 (5.6%)	0.206
Irritative or pain requiring medicine > 7 days	0	4 (5.6%)	0.057
Grade II (early)			
Re-catheterization	7 (9.5%)	7 (9.9%)	1.000
Hematuria clot retention	1 (1.4%)	5 (7.0%)	0.113
Bleeding requiring transfusion	0	2 (2.8%)	0.246
Epididymo-orchitis	2 (2.7%)	2 (2.8%)	1.000
Grade II (late)			
Hematuria	2 (2.7%)	6 (8.4%)	0.163
Recurrent UTI	2 (2.7%)	5 (7.0%)	0.272
Urge incontinence	2 (2.7%)	5 (7.0%)	0.272
Stress incontinence	0	0	—
Dysuria or perineal pain	1 (1.4%)	4 (5.6%)	0.206
Grade IIIa (late)			
Urethral stricture	5 (6.8%)	5 (7.0%)	1.000
Urinary retention	0	0	—
Grade IIIb (late)			
Re-operation	1 (1.4%)	5 (7.0%)	0.113
Grade IVa			
Urosepsis causing circulatory failure	0	0	—
Grade IVb			
Cardiopulmonary failure requiring ICU care	0	0	—
TUR syndrome requiring ICU care	0	1 (1.4%)	0.493
Death	0	0	—
Sequela			
Retrograde ejaculation**	5/23 (21.7%)	7/24 (29.2%)	0.740

*Statistical difference.

**Only patients with sexual activities were analyzed.

No statistically significance was observed between two groups in urinary tract infection (UTI), re-catheterization, clots retention, epididymo-orchitis, urinary incontinence and TUR syndrome within 30 days. The rate of late complications was comparable between two groups, including hematuria, dysuria, urethral stricture, urge incontinence, and stress incontinence. We observed a higher re-operation rate with the PKRP group (7.0% vs. 1.4%, *p* = 0.113). The causes of re-operation in PKRP group were two cases with bladder neck contracture, one with residual adenoma, and two with urethra stricture. There was a case undergoing MPVP developed a bladder neck stone 11 months postoperatively, and removal of the stone was done.

## Discussion

In the past two decades, a wide range of innovative transurethral procedures have challenged the standard surgical treatment, monopolar TURP, in the treatment of BPO^12^. PKRP has efficacy similar to TURP with fewer adverse events^13^. Laser prostatectomy techniques such as holmium laser enucleation of the prostate (HoLEP), Green Light photoselective vaporization of the prostate (PVP) and thulium laser resection of the prostate (ThuRP), have been widely adopted in clinical practice for the comparable or improved safety and efficacy with monopolar TURP^1,2,14^.

Several studies demonstrated that thulium laser intrinsically possesses several advantages, such as more efficient operation, improved spatial beam quality and more precise tissue incisions^7,15,16^. Different thulium laser techniques have been described, including thulium vapoenucleation (ThuVEP), thulium laser vaporesection of the prostate (ThuVaRP)^17^, thulium laser enucleation (ThuLEP), and thulium vaporization (ThuVP)^18^. ThuVaRP provides efficient resection and vaporization at the same time, and thus a faster tissue ablation rate^14^. Several studies have described the advantages and disadvantages of various laser prostatectomy techniques such as thulium vaporesection^7,15,16^, thulium laser enucleation^18^, diode laser vaporesection^19–22^, and Green Light laser vasporization^5^.

**Figure 2:**
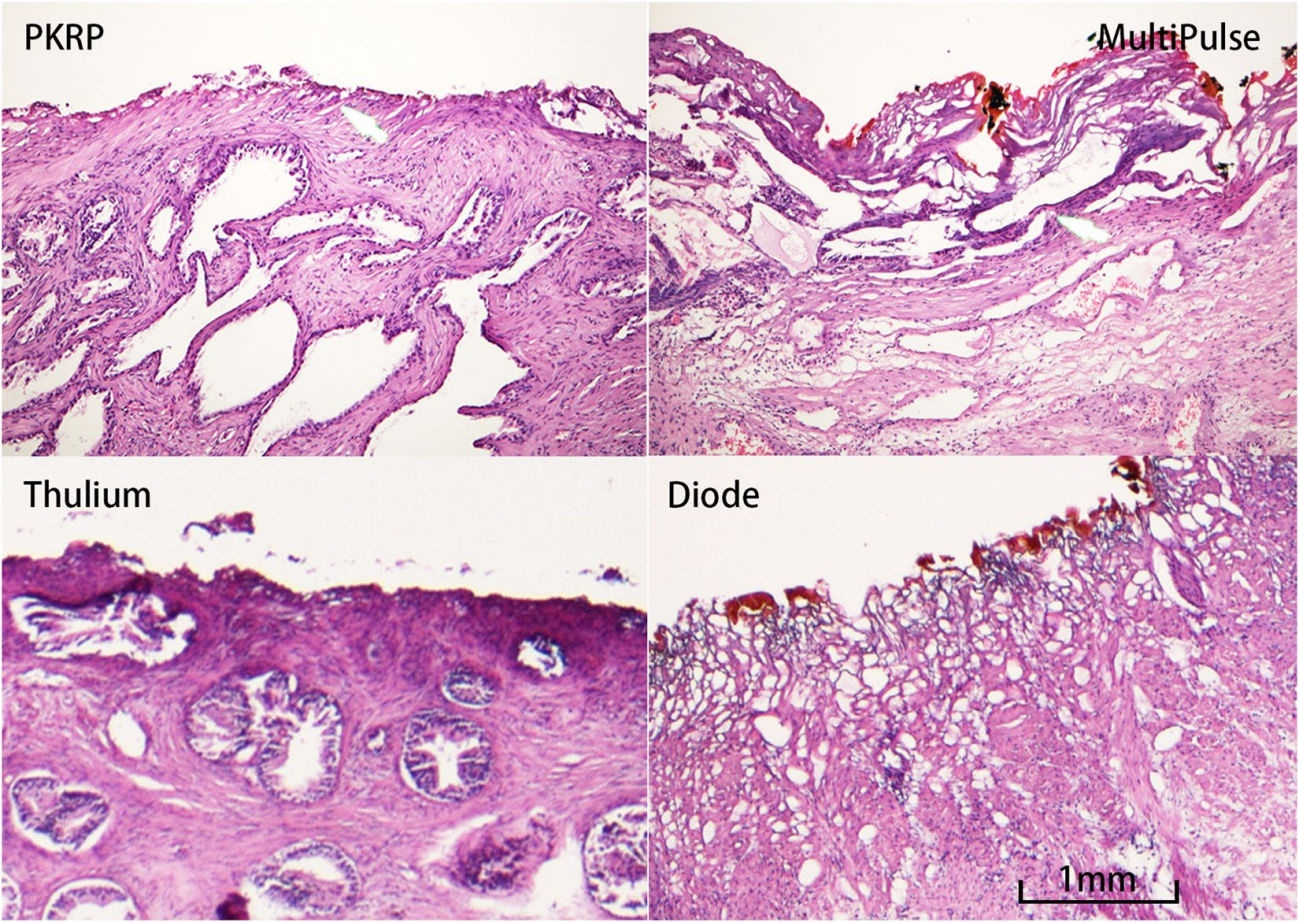
Thermal injury of different kinds of lasers and plasmakinetic resection of the prostate. Under haematoxylin and eosin (H&E) stain. The magnifying power was 200X.

Most investigators agreed that, the extent of thermal damage is associated with hemostasis. Several previous studies analyzed the coagulation zone for different laser prostatectomy techniques to evaluate thermal damage PKRP has a coagulation layer of 0.3–1.0 mm, while thulium laser provides a 0.5–2.0 mm coagulation zone^23,24^. In animal models, the application of a 980 nm 150 Watts diode laser for prostate vaporization resulted in a necrotic zone of 6.1 ± 1.2 mm^25^, while the 120 Watts Green Light laser showed a 1.5 ± 0.3 mm coagulation band^26^. In the present study, we compared tissue thermal damage with PKRP, Multipulse, thulium and diode lasers in our patients (Fig. 2). The depth of thermal damage was 0.2–0.5 mm in PKRP, 0.5–1.0 mm in thulium laser (LISA Laser Products OHG, Katlenburg-Lindau, Germany), 0.5–1.5 mm in Multipulse laser, 2–4 mm in diode laser (Limmer Laser GmbH, Berlin, Germany), respectively. The coagulation zone of Multipulse laser was between those of thulium and diode laser. Consequently, the hemostatic efficacy of Multipulse laser was relatively superior to plasmakinetic prostatectomy.

Consistently, the blood transfusion rate related to perioperative bleeding was lower in the MPVP group (0 vs. 2.8%), without statistical significance (*p* = 0.246). The excellent hemostatic effect of Multipulse laser resulted in shorter catheterization time and hospital stay in our study.

In our study, the operative time was shorter in MPVP group (30.9 ± 10.3 vs. 36.4 ± 13.8 minutes, *p* = 0.008).The excellent hemostatic efficacy of MPVP provided a nearly bloodless operation field during the procedure. Two experienced surgeons could adequately resected prostate chip to reduce the use of morcellator, allowed operative time to be shortened. In contrast, it took more time for hemostasis in PKRP procedure. Additionally, the weight of the resected prostate tissue was heavier in the PKRP group (11.0 ± 8.0 vs. 20.6 ± 10.5 gm, *p* < 0.001). However, there was no significant difference in residual prostate volume ratio at 12 months between the two groups. Previous study demonstrated that the thulium laser concomitantly vaporized prostate tissue during cutting^27,28^, which could further shorten the operative time.

Yang reported a randomized study to compare ThuLEP with PKRP^18^. Both procedures did not differ significantly in terms of Qmax, IPSS, PVR, and QoL through 18 months of follow-ups. A meta-analysis showed no significant difference in IPSS, QoL, PVR and Qmax between ThuVaRP and with TURP or PKRP during the 3-, 6-, and 12-month follow-ups^14^. Our study showed no significant difference in IPSS, Qmax, residual prostate volume ratio, IIEF-5 and PSA drop between MPVP and PKRP groups at the 12-month follow-up (Table 3). Nevertheless, after mixed model analysis with random effect and Bonferroni correction, MPVP group was superior to PKRP group in IPSS and QoL (*p* = 0.049 and <0.001, respectively). These finding could be explained by the significant differences between both groups in IPSS and QoL at 1 month postoperatively (Fig. 1). Similar results have been reported by Deng *et al*. through a systematic review and meta-analysis on ThuVaRP versus PKRP^14^. The better QoL may have arisen from the lower overall complication rate in the MPVP group.

Both PKRP or MPVP procedures were performed with normal saline irrigation and overcame TURP’s well-known disadvantages. Nevertheless, there was one patient (1.4%) who developed fluid overloading and respiratory distress after PKRP (grade IVa complication). There was no grade IVa complications in the MPVP group.

A meta-analysis revealed that thulium vaporesection did not differ significantly from bipolar TURP in terms of complications of urethral stricture, urge incontinence, bladder neck contracture and blood transfusion^14^. In our study, most postoperative complications in the MPVP group were mild and occurred within first 30 days: urinary tract infection (UTI) and re-catheterization (6.8% and 9.5%, respectively). Conversely, grade II complications in the PKRP group were higher: clot retention, persistent hematuria, recurrent UTI, and dysuria or perineal pain (7.0%, 8.4%, 7.0% and 5.6%, respectively; *p* > 0.05). There were 5 cases (7.0%) in the PKRP group that developed bladder neck contracture, residual adenoma or urethra stricture requiring re-operation (grade IIIb complications); only one patient needed re-operation in the MPVP group. The overall complication rate was significantly higher in the PKRP group (45.0% vs. 21.9%, *p* = 0.004).

There were several limitations in this study. First, the case number was limited. The power analyses showed the power of the primary outcomes, IPSS and Qmax, was strong enough to detect a significant difference, instead of the other two variables, PVR and QoL. This results could be a reference for the randomized trial in the future. Second, a lack of randomization may compromise the results in view of evidence medicine; thus, patients selection bias dose exist. Third, surgeon’s experience may significantly affect the outcomes, especially the complications. The two surgeons in this study were board-certificated urologist and had more than eight years of experience in performing transurethral prostatectomies with laser or electrocauteries in over 800 cases.

## Conclusion

MPVP and PKRP provided comparable symptomatic improvements. MPVP, combining thulium and diode laser energy, showed better hemostasis, shorter catheterization time, shorter hospital stay and less complications compared to PKRP. It is warranted to conduct a randomized controlled study with more case number to validate these findings limited by selection bias and statistical power.

### Compliance with ethical standards

**Disclosures** Drs Fu-Shun Hsu, Chen-Wei Chou, Hong-Chiang Chang, Yuan-Po Tu, Shing-Jia Sha, Huang-Hsin Chung, and Kuo-How Huang have no conflict of interest or financial ties to disclose.
