# Author Correction: Comparison of Multipulse Laser Vaporesection versus Plasmakinetic Resection for Treatment of Benign Prostate Obstruction

**DOI:** 10.1038/s41598-019-54254-3

**Published:** 2019-11-25

**Authors:** Fu-Shun Hsu, Chen-Wei Chou, Hong-Chiang Chang, Yuan-Po Tu, Shing-Jia Sha, Huang-Hsin Chung, Kuo-How Huang

**Affiliations:** 1 Department of Urology, New Taipei City Hospital, New Taipei City, Taiwan; 2 Graduate Institute of Clinical Medicine, National Taiwan University College of Medicine, Taipei, Taiwan; 3 Department of Urology, National Taiwan University Hospital, Taipei, Taiwan; 4 Shu-Tien Urology Ophthalmology Clinic, Taipei, Taiwan; 5 Department of Pathology, New Taipei City Hospital, New Taipei City, Taiwan

Correction to: *Scientific Reports* 10.1038/s41598-019-42903-6, published online 23 April 2019

The original version of this Article contained a typographical error in the spelling of the author Kuo-How Huang, which was incorrectly given as Kou-How Huang. This has now been corrected in the PDF and HTML versions of the Article.

